# Intraoperative Serum Catecholamine Levels in a Pregnant Woman With Pheochromocytoma Undergoing Cesarean Delivery With Combined Spinal-Epidural Anesthesia: A Case Report

**DOI:** 10.7759/cureus.24727

**Published:** 2022-05-04

**Authors:** Haruna Miyazaki, Daisuke Miura, Yukie Koguchi, Chihiro Takamatsu, Yoshiro Sakaguchi

**Affiliations:** 1 Department of Anesthesiology, Saga-Ken Medical Centre Koseikan, Saga, JPN; 2 Department of Anesthesiology, School of Medicine, Saga University, Saga, JPN; 3 Department of Anesthesiology, Takagi Hospital, Fukuoka, JPN

**Keywords:** spinal anesthesia, serum catecholamine levels, pheochromocytoma, epidural anesthesia, cesarean delivery, case report

## Abstract

Pheochromocytoma has a significant effect on perioperative hemodynamics; however, little is known about the changes caused by pheochromocytoma in pregnant women during cesarean delivery. Moreover, cesarean delivery in pregnant women with pheochromocytoma is often performed, along with pheochromocytoma removal, under general anesthesia depending on the time of delivery. Therefore, changes in the hemodynamics of these patients during cesarean delivery under spinal anesthesia combined with epidural anesthesia, along with their serum catecholamine concentration, have not been reported.

In this report, we describe the changes in the maternal intraoperative hemodynamics and serum catecholamine level of a pregnant woman with pheochromocytoma who underwent cesarean delivery under combined spinal-epidural anesthesia at 35 weeks of gestation. No significant change in the hemodynamics and serum catecholamine level was observed, and the procedure was carried out safely.

Cesarean delivery in an optimized pheochromocytoma patient under combined spinal-epidural anesthesia might be feasible without concurrent surgical removal of pheochromocytoma.

## Introduction

Pheochromocytoma occurs in only 0.002% of pregnancies [1,2]. Further, its diagnosis is difficult because the variable clinical presentation makes it challenging to suspect the condition, and both the mother and the child are at risk of perinatal mortality if not managed properly during the pregnancy [3]. Several reports have demonstrated anesthesia management for cesarean delivery in pregnant women with pheochromocytoma. Moreover, various reports on general and regional anesthesia for surgery, not limited to cesarean delivery, in patients with pheochromocytoma have been reported [4-7]. Although regional anesthesia is safer for the mother and the fetus during cesarean delivery [8], the optimal anesthesia for cesarean delivery in pregnancies complicated by pheochromocytoma has not been established [9], and no cases involved intraoperative measurement of serum catecholamine levels.

Here, we present the case of a pregnant woman with pheochromocytoma who underwent cesarean delivery with combined spinal-epidural anesthesia (CSEA) and the intraoperative measurement of serum catecholamine level. The patient provided written consent for the publication of this case report. This article adheres to the CARE guidelines.

## Case presentation

A 37-year-old woman (height 162 cm, weight 54 kg), primigravida, with no significant medical or family history, was admitted by her previous doctor for the management of gestational hypertension at 29 weeks and four days of gestation. At 30 weeks and three days of gestation, the patient experienced marked nocturnal sweating and tachycardia, and transabdominal ultrasonography revealed a mass in the right adrenal gland. She was transferred to the Department of Obstetrics and Gynecology at our hospital the next day with suspicion of a pregnancy complicated by pheochromocytoma. Close examination revealed elevated blood catecholamine levels, elevated urinary catecholamine levels, and elevated urinary levels of vanillylmandelic acid, metanephrine, and normetanephrine (Table 1). Abdominal magnetic resonance imaging revealed a 4-cm mass in the right adrenal gland, leading to the diagnosis of pregnancy complicated by pheochromocytoma. At 30 weeks and four days of gestation, according to the preoperative management of pheochromocytoma resection [10], 2 mg doxazosin mesylate was started, and the dosage was gradually increased to 16 mg/day over one month and continued for nine days until the surgery. Based on the plasma renin activity of 91.8 ng/mL/hour and serum aldosterone level of 2330 pg/mL at admission, the patient’s circulating blood volume was considered inadequate, and daily fluid infusion was administered (approximately 1,000 mL of normal saline/day) from five days before the day of the surgery. Preoperatively, the plasma renin activity and serum aldosterone concentration had corrected to 8.9 ng/mL/hour and 605 pg/mL, respectively.

**Table 1:** Laboratory findings. Laboratory findings show elevated blood catecholamine levels, elevated urinary catecholamine levels, and elevated urinary levels of vanillylmandelic acid, metanephrine, and normetanephrine.

	Patient value	Reference range
Blood catecholamine		
Adrenaline	370	<100 pg/mL
Noradrenaline	7,900	100–450 pg/mL
Dopamine	120	<20 pg/mL
Urinary catecholamine		
Adrenaline	103	1.1–22.5 μg/day
Noradrenaline	2,020	29.7–118.0 μg/day
Dopamine	1,300	100–1,000 μg/day
Vanillylmandelic acid	13.2	1.5–4.3 mg/day
Metanephrine	0.38	0.04–0.19 mg/day
Normetanephrine	3.42	0.09–0.33 mg/day

Cesarean delivery was performed at 35 weeks and two days of gestation, and adrenal tumor resection was planned for a later date. The preoperative blood pressure was well controlled, and no symptoms of a hypertensive crisis were observed. Considering the child’s safety, CSEA was selected as the method of anesthesia, and general anesthesia would be induced in case of an emergency. The infant was suctioned to avoid upper abdominal compression during delivery.

**Figure 1:**
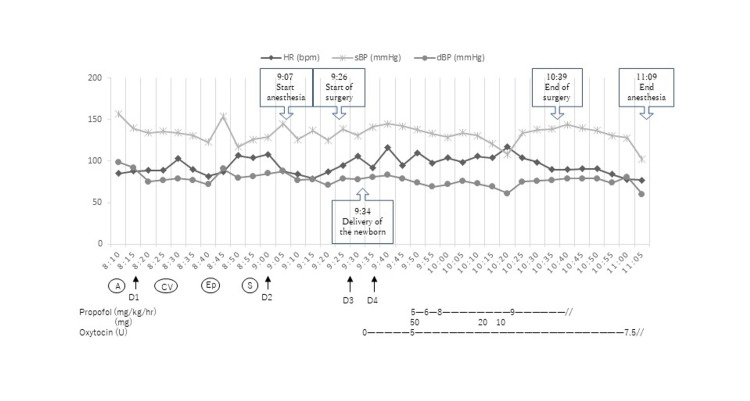
Anesthesia record of the patient. HR: heart rate; sBP: systolic blood pressure; dBP: diastolic blood pressure; A: indwelling arterial pressure catheter; CV: indwelling central venous pressure catheter; Ep: indwelling epidural catheter; S: spinal anesthesia; D1: the timing of blood sampling one (before induction of anesthesia); D2: the timing of blood sampling two (from the induction of anesthesia to the operation); D3: the timing of blood sampling three (time of newborn delivery); D4: the timing of blood sampling four (after delivery).

The anesthesia process is shown in Figure 1. After securing the arterial and central venous lines under local anesthesia, an epidural catheter was inserted through the T12/L1 space. Spinal subarachnoid anesthesia was administered through L3/4, and bilateral analgesia below the T5 level was obtained with 14 mg bupivacaine. The initial dose of bupivacaine was determined to be 12 mg to obtain adequate analgesia. An additional 1 mg was added three minutes later, as adequate hypesthesia was not obtained, and another 1 mg was added after four additional minutes. After delivery, a total of 7.5 units of oxytocin was administered, and the patient was sedated with propofol to relieve her anxiety after meeting her newborn. Intraoperative hemodynamics were stable without the use of vasoactive agents.

The anesthesia time was 122 minutes, and the operative time was 73 minutes. The volume of fluid infusion was 3,254 mL, the volume loss (including blood and amniotic fluid) was 1,204 mL, and the volume of urine produced was 700 mL. The male newborn had a birth weight of 2,446 g and a cord blood pH of 7.30. Apgar scores at one and five minutes of life were 8 and 10 (-1 for skin color and respiration, each). The changes in blood catecholamine levels measured intraoperatively are shown in Table 2. Blood samples were collected from the arterial pressure line preoperatively, before the induction of anesthesia, induction of anesthesia for surgery, delivery of the newborn, and after delivery. All these measurements were taken postoperatively. Although there was a slight increase in the noradrenaline level to 9.20 ng/mL immediately following delivery, no hypertension was observed.

**Table 2:** Intraoperative blood catecholamine levels. The changes in blood catecholamine levels measured intraoperatively.

Catecholamine (pg/mL)	Preoperative	Before anesthesia induction	Induction of anesthesia for surgery	Delivery of newborn	After delivery
Adrenaline (<100)	370	130	110	80	130
Noradrenaline (100–450)	7,900	6,900	5,300	5,300	9,200
Dopamine (<20)	120	50	50	50	80

After the surgery, the patient was admitted to the intensive care unit and then moved to the general ward the next day without difficulties. Approximately one month after cesarean delivery, she underwent removal of the adrenal tumor.

## Discussion

Pheochromocytoma occurs in one per 15,000 to 54,000 pregnancies [3]. The mother is at risk of hypertensive crisis, cerebrovascular accident, and arrhythmia; in addition, the fetus is at risk of placental abruption and intrauterine hypoxia because of the vasoconstrictive effects of catecholamines [1-3]. Studies before 1970 demonstrated that without early diagnosis and proper management of pheochromocytoma, maternal and fetal mortality risks as high as 48% and 54%, respectively, are conferred [11]. However, recent systematic reviews have reported maternal and fetal mortality rates of 8% and 17%, respectively, with antepartum diagnosis and appropriate treatment [12].

In healthy pregnant women, the plasma and urinary catecholamine levels are not consistently elevated [3,12]. However, the intra-abdominal pressure (IAP) increases consistently during pregnancy, reaching over 13 mmHg after 32 weeks of gestation [13]. Previous studies in laparoscopic surgery have demonstrated that catecholamine secretion increases when IAP exceeds approximately 15 mmHg [14]. In addition, in patients with pheochromocytoma undergoing laparoscopic adrenalectomy, pneumoperitoneum induced a significant increase in serum catecholamine concentrations, especially with surgical stress during manipulation of the adrenal gland, which also promoted a significant increase [15]. This suggests that in pregnant women with catecholamine-producing tumors, maternal circulating catecholamine levels may rise and symptoms may worsen owing to the growing uterus, fetal movement, uterine contractions, and palpation of the abdomen [3,16]. Although a cesarean delivery should be performed as soon as possible before maternal risk increases, a sufficient amount of fetal pulmonary surfactant is not produced until after 34 weeks of gestation. Therefore, after 32 weeks of gestation, it is preferable to wait for fetal maturation until 34 weeks of gestation, when the lung surfactants are produced in sufficient quantities and breathing is possible, to enable better fetal outcome.

Laparoscopic removal of the tumor during pregnancy is recommended if it is diagnosed before 24 weeks of gestation [3,12]. If diagnosed after 24 weeks, it is advisable to first perform a cesarean delivery and remove the tumor at the same time or later owing to the difficulty in accessing the tumor [3,12,16]. Removing the tumor at the same time requires general anesthesia and is risky for the fetus.

There are no data supporting either cesarean delivery or vaginal delivery as the recommended delivery method in pregnant women with pheochromocytoma [3]. In previous cases, vaginal delivery had a higher maternal mortality rate than cesarean delivery, and for many years, cesarean delivery was preferable [11,17]. However, in contemporary obstetric and anesthetic management, vaginal delivery under adequate epidural anesthesia has been safely and successfully performed in some cases; thus, the debate on the optimal method of delivery continues [18].

The optimal anesthesia for cesarean delivery in pregnancies complicated by pheochromocytoma has not been established [9]. In general anesthesia, analgesia with sufficient blockade of afferent input, as with the use of high-dose remifentanil, is necessary due to the hemodynamic changes induced. Hypertension should be prevented and treated with vasodilators [15,19]. However, no agent can completely prevent the hemodynamic changes. Regional anesthesia is advantageous because it blocks the afferent pain signals and the central stimuli to the adrenal glands, preventing a sympathetic nervous system-mediated catecholaminergic response. However, even minor stimuli, such as abdominal pressure and postural changes during regional anesthesia, may induce remarkable hypertension and tachycardia [20]. Hypotension may also develop owing to decreased circulating plasma volume.

In the present case, the preoperative blood noradrenaline level was high due to pheochromocytoma and did not increase with anesthesia or surgery up to the delivery of the newborn. A mild increase was observed following delivery; however, no hypertension was observed. The patient did not complain of intraoperative pain or discomfort, and we assumed that the sympathetic block through spinal subarachnoid anesthesia was sufficient to suppress catecholamine release. Although the bupivacaine dosage could have been reduced, priority was given to obtaining a sufficient anesthetic range to exclude the hemodynamic changes associated with pain stimulation due to inadequate hypesthesia and the hemodynamic changes due to catecholamine release from the tumor. Therefore, in pregnancies complicated by pheochromocytoma, which do not require tumor manipulation, and the preoperative blood pressure is adequately controlled by adequate alpha blockade and circulating blood volume is optimized, regional anesthesia can be chosen over general anesthesia for cesarean delivery.

## Conclusions

There are no previous studies reporting changes in catecholamine levels during cesarean delivery in pregnant women with pheochromocytoma. This case is significant because it demonstrates the successful anesthetic management of a cesarean delivery with CSEA that did not affect intraoperative catecholamine levels. Anesthesia management for cesarean delivery in pregnant women with pheochromocytoma should be examined on a case-by-case basis, considering the preoperative blood pressure control, timing of cesarean delivery, and simultaneous removal of pheochromocytoma. The case was managed safely; however, future prospective studies on the safety of cesarean delivery in patients with pheochromocytoma under CSEA are necessary.
